# Hippocampal activity during the transverse patterning task declines with cognitive competence but not with age

**DOI:** 10.1186/1471-2202-11-113

**Published:** 2010-09-08

**Authors:** Vera M Leirer, Christian Wienbruch, Isabella Paul-Jordanov, Stephan Kolassa, Thomas Elbert, Iris T Kolassa

**Affiliations:** 1 Clinical Psychology & Neuropsychology, University of Konstanz, Universitätsstr. 10, 78457 Konstanz, Germany; 2 Zukunftskolleg, University of Konstanz, Box X 916, 78457 Konstanz, Germany; 3 Research & Innovation, SAF Simulation, Analysis & Forecasting AG, Bahnstr. 1, 8274 Tägerwilen, Switzerland

## Abstract

**Background:**

The hippocampus is a brain region that is particularly affected by age-related morphological changes. It is generally assumed that a loss in hippocampal volume results in functional deficits that contribute to age-related cognitive decline. In a combined cross-sectional behavioural and magnetoencephalography (MEG) study we investigated whether hippocampal-associated neural current flow during a transverse patterning task - which requires learning relational associations between stimuli - correlates with age and whether it is modulated by cognitive competence.

**Results:**

Better performance in several tests of verbal memory, verbal fluency and executive function was indeed associated with higher hippocampal neural activity. Age, however, was not related to the strength of hippocampal neural activity: elderly participants responded slower than younger individuals but on average produced the same neural mass activity.

**Conclusions:**

Our results suggest that in non-pathological aging, hippocampal neural activity does not decrease with age but is rather related to cognitive competence.

## Background

Normal aging is accompanied by a multitude of morphological changes in the brain. Consistently, volumetric MRI studies show a decline in total brain volume due to aging [e.g. [1-7]]. Although brain shrinkage is relatively widespread, brain regions differ in their intensity of volume loss [e.g. [8,9]]. Longitudinal [e.g. [8,10]], as well as cross-sectional studies [e.g. [11]] demonstrate that especially the caudate, the cerebellum, tertiary association cortices and the hippocampus are affected by shrinkage. Furthermore, Raz et al. [8] report that particularly in the hippocampus - essential for declarative memory, spatial navigation and contextual relations [12,13] - the magnitude of shrinkage accelerates with increasing age: the hippocampus shrinkage rate in healthy older people is more than twice that of younger people [1,14-16]. Volume reductions within the hippocampus were also analyzed in a recent MRI study with volume reduction found to be more severe in posterior regions [17]. In sum, these observations demonstrate that the hippocampus is structurally especially affected by the aging process. The loss may be pronounced in pathological ageing, e.g., in Morbus Alzheimer the hippocampus is one of the regions being particularly vulnerable to degeneration [18].

Morphological changes, however, are not linearly related to brain function. Nonetheless, it is plausible to assume that regional brain damage will alter neural firing patterns in space and time. Hence, age-related changes in neural activation during memory tasks should be expected in the medial temporal lobe, notably the hippocampal region. On this account, several studies employing functional neuroimaging methods examined age effects on neuronal activation patterns during diverse memory tasks [e.g. [19-30]]. Results concerning age effects on activity of the hippocampal region differ across studies. Some studies report more activity in elderly people in right parahippocampal regions during memory retrieval [20], in the right hippocampus during autobiographical memory tasks [30] as well as higher cerebral blood flow increase in the medial temporal lobe during memory encoding [29]. In contrast, there are also studies that report less activation for elderly people in mediotemporal structures, including the hippocampus and the parahippocampus, during encoding [21,24], encoding and retrieval [19], correct word recognition [27], working memory tasks [25,26] as well as during visual attention and episodic retrieval [26]. Furthermore, there are also a number of studies that do not find age-related differences in the magnitude of activation in the hippocampal region during memory encoding [22,23,28,31-33].

Given that the hippocampus is thought to be responsible for the organization and structuring of memory storage [34,35] rather than for keeping the code of memory contents, this might explain why the results of studies concerning age-related differences in mediotemporal activation patterns during memory tasks are rather inconsistent. In the present study we therefore employed the transverse patterning task [36,37], i.e., contextual processing which consistently engages hippocampal function.

The transverse patterning paradigm requires learning nontransitive binary relations between three stimuli, pairs of which are presented. The well-known game "rock-paper-scissors" is an example of a transverse patterning paradigm with meaningful stimuli. The problem cannot be solved with elemental discriminations of single stimuli but requires learning relational associations [37,38]. The hippocampal formation is thought to play a central role in configural learning [12,13]. Consistent with this theory are the findings that damage of the hippocampal formation in humans [39-41], rats [37] and monkeys [42-44] impairs performance on the transverse patterning task. Furthermore, Driscoll and colleagues [45] found age-related differences in behavioral performance on a transverse patterning task: compared to younger people, elderly people were less often able to complete the transverse patterning task successfully, required more trials to reach criterion and made more mistakes. Here, we used magnetoencephalography (MEG) to track the fast neural responses induced by the transverse patterning task. A series of studies has demonstrated that activity from the medial temporal lobe can be assessed with MEG [46-63].

We specifically planned on assessing whether the hippocampal-associated activation pattern in healthy people changes with age. Furthermore, we recorded the extent to which this neural activity is associated with cognitive competence.

## Methods

### Participants

Fifty-two right-handed and healthy subjects (23 males and 29 females) ranging in age from 18 to 89 years (*m *= 52.4 years, *sd *= 19.6) (Additional file 1) with a mean MMSE score of 29.4 (SD = 1.08) participated in this study. Their mean education was 15 years (ranging from 10 to 22 years). None of the included participants reported a history of drug and/or alcohol abuse. Subjects were recruited by notifications posted on the campus of the University of Konstanz and in several residential homes for the elderly in the Konstanz area and by advertisement in the local newspaper and radio station. They were paid 30€ for their participation. Exclusion criteria were acute as well as a history of psychiatric diseases, a history of psychopharmacological medication, left-handedness (to control for potential influences of brain lateralization due to handedness), metal objects in the body as well as a history of severe head injuries or neurological problems (like epilepsy, strokes, brain tumors etc.). The ethics committee of the University of Konstanz approved this study.

### Procedures

Upon arrival at the laboratory, participants were familiarized with the MEG chamber, and study procedures and goals were clarified. All participants gave written informed consent. Afterwards subjects were screened with the MINI International Neuropsychiatric Interview [64] to assure that the participants did not suffer from a psychiatric disease. Subsequently, demographic data were assessed and handedness was determined using the Edinburgh Inventory [65]. Furthermore, cognitive abilities were assessed with the *CERAD-NP-plus *test battery [66] with the subtests Verbal Fluency (VF = sum score of semantic and phonemic fluency), Word List Learning (WLL), Word List Delayed Recall (WLDR), Word List Recognition (WLR), Figure Recall (FR), Trail Making Test A and B (TMT-A/B). Additionally, the Digit Symbol and the Digit Span subtests of the German version of the Wechsler Adult Intelligence Scale [HAWIE-R; [67]] as well as the *Benton Visual Retention Test *- *revised form *[68] were conducted.

#### Training

**Figure 1:**
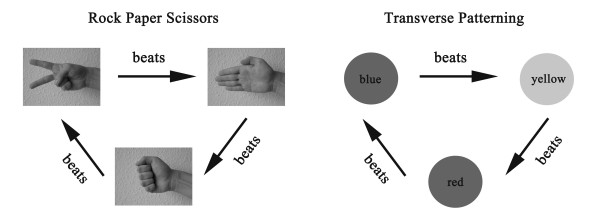
**Stimuli of the Rock, Paper, Scissors (S+P-, P+R-, R+S-) and the Transverse Patterning (B+Y-, Y+R-, R+B-) task**.

The two tasks and the basic design that were used in this experiment were adapted from the study of Hanlon and colleagues [50,51]: the *Rock, Paper, Scissors *task (RPS) and the *Transverse Patterning *task (TP) (Figure 1). The well-known childhood game "Rock, Paper, Scissors" is an analogous task to the TP task with meaningful stimuli and relations. It was chosen as a training task to familiarize subjects with the underlying logic of transverse patterning designs.

Immediately prior to the MEG measurement, participants were trained on both tasks to familiarize themselves with the procedure. The first task to train was RPS where two out of three pictures of a hand symbolizing rock, paper or scissors were presented simultaneously. The significance of any one item depends on with which other item it is currently paired: When rock and scissors are paired, rock is correct, when scissors and paper are paired, scissors are correct, and if paper and rock are presented together, paper is correct (R+ S-, S+ P-, P+ R-).

In the second task (TP), the stimuli consisted of three disks in the colors blue, yellow or red. To solve the task, participants had to learn the following rule: When blue and yellow are presented together, blue is correct, when yellow and red are presented together, yellow is designated correct, and when red and blue are shown together, red is correct (B+ Y-, Y+ R-, R+ B-) (Figure 1). Participants learned the abstract transverse patterning rule through explicit instruction.

In each trial of the RPS and TP task, two of the three pictures were presented next to each other on a grey computer screen using Presentation^® ^(Neurobehavioral Systems, version 9.00). All stimuli were 9 × 10 cm (height × width). Participants were instructed to choose the correct picture as fast as possible by pressing the corresponding mouse button (left button for the left picture and right button for the right picture). The button press cleared the stimulus pair from the screen. Pictures were balanced for left and right side presentation.

**Figure 2:**
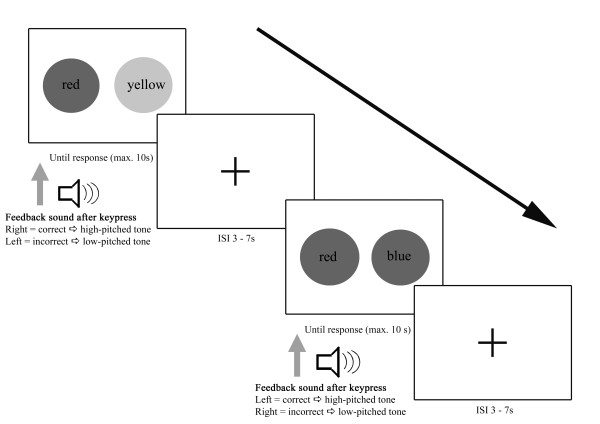
**Experimental design of the Transverse Patterning task**.

Each trial began with the presentation of a grey screen with a fixation cross for 3-7 s to aid participants in maintaining gaze on the center of the screen, followed by the presentation of the stimulus pairs. Each pair of stimuli was presented for up to 10 s or until the participant responded via mouse button press. The response was followed immediately by a feedback sound: a high-pitched tone (1000 Hz) if the reaction was correct and a low-pitched tone (250 Hz) if the answer was incorrect. If the subject did not respond after 10 s, the trial was considered incorrect and the picture disappeared, the fixation cross appeared, and a new trial started (Figure 2). Participants either needed to accomplish 10 correct training trials in a row or 42 trials altogether.

#### MEG testing

After training, MEG recordings were obtained, first for the RPS task to familiarize subjects with the MEG environment and then for the TP task. For artifact rejection, we recorded the electro-occulogram (EOG) using bipolar electrode configuration where electrodes were attached near the left and right outer canthus and below and above the right eye. For recording of the electrocardiogram (ECG), two additional electrodes were attached at the left lower forearm and the right collarbone. Subsequently, participants were seated in the magnetically shielded room (Vakuumschmelze Hanau) and their head shapes were digitized with a Polhemus 3 Space Fasttrack (Polhemus, Colchester, VT, USA). Five index points were determined to calculate the relative head position within the MEG sensor for source analysis. The subjects' head position relative to the pickup coils of the sensor was estimated before and after the measurement. During MEG measurement subjects were lying supine in a comfortable position. A video camera installed inside the chamber allowed monitoring subjects' behavior and ensured compliance throughout the experiment. To avoid artifacts, subjects were asked to stay still and to avoid head and eye movements as well as eye blinks during trials.

The experimental set-up and the stimuli were similar to the training procedure. Pictures were presented with a video projector (JVC™, DLA-G11E) with a refresh rate of 100 Hz on a white plastic screen attached to the ceiling of the room. Subjects responded by pressing the corresponding button on a button box with their right hand. In order to avoid magnetic interference, the feedback tones were presented through ear tubes. Sound intensity was adapted to the individual hearing level before measurement. MEG was recorded continuously and digitized at a rate of 678.17 Hz using a 148-channel whole head magnetometer (MAGNES™, 2500 WH, 4 D Neuroimaging, San Diego, USA). A real band-pass filter of 0.1 - 200 Hz was used for data acquisition. EOG and ECG were recorded with a SynAmps amplifier (Neuroscan ™) using Ag/AgC1 electrodes.

The experiment ended either after the subject had achieved 120 correct trials or when 180 trials in total had been presented.

### Data Analysis

**Figure 3:**
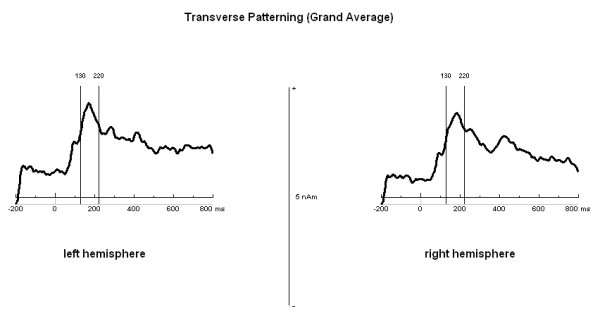
**Source waveforms of the hippocampal sources during the transverse patterning task**. Source waveforms (grand average of the correct trials over all subjects) of the hippocampal sources for the left and right hemispheres during the transverse patterning task. The marked time segments of 130-220 ms after stimulus presentation were chosen for further analyses.

**Figure 4:**
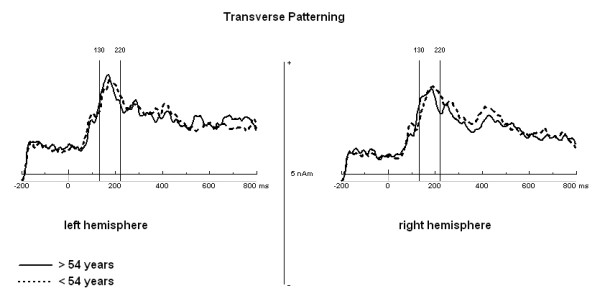
**Source waveforms of the hippocampal sources for two age groups found by median split**. Source waveforms of the hippocampal sources for the left and right hemispheres during the transverse patterning task plotted separately for the two age groups found by median split (median: 54,5 years; young group: 18-54 years; old group: 55-89 years).

Since the RPS task was chosen as a training task in order to familiarize subjects with the transverse patterning design and the MEG environment, data analysis was solely conducted for the transverse patterning task. After noise-reduction of environmental magnetic noise (noise reduction procedure, 4 D Neuroimaging), using the reference channels of the WH2500 data were analyzed using BESA (Brain Electrical Source Analyses; MEGIS Software GmbH) software version 5.2.4.. Cardiac activity and eye movements were removed with a semi-automated procedure implemented in BESA. For each subject, epochs with a 200 ms baseline and a post trigger (stimulus presentation) window of 800 ms were generated. After baseline correction, a bandpass-filter of 1-40 Hz (Low Cutoff filter: 1 Hz, forward, 6 dB/oct; High Cutoff filter: 40 Hz, zero phase, 24 dB/oct) was applied to the resulting data epochs. Afterwards, data epochs were averaged separately for correct and incorrect trials. In order to transform surface MEG into brain source activity, a source montage with 51 fixed regional dipoles in a homogeneous sphere was defined [69]. In the case of MEG a regional dipole consists of two perpendicular, fixed equivalent current dipoles describing the two tangential components of a source, while the radial component does not contribute to the magnetic field outside the volume conductor. As we were interested in brain activity originating from hippocampal regions, the source montage consisted of 2 regional dipoles (Talairach coordinates: x = ± 42.4; y = -9.5; z = -23.9 for the right/left hemisphere) for assessing activity from the medial temporal lobes and 49 other regional dipoles that represented other brain areas, thus increasing the sensitivity of the fixed sources that had been positioned in the hippocampal region. All dipoles of the source montage were set by hand with fixed location and orientation in similar distance to the head surface to minimize differences in the sources' explanation of variance. In order to explore the time window for further analyses of dipole strength, a grand average of the correct trials of all subjects was generated. The time segment of 130-220 ms after stimulus presentation, containing the peak amplitude of the hippocampal source waveforms in both hemispheres, was chosen for further analyses (Figure 3 and 4). After selecting the time window of interest, dipole analysis was carried out on each participant. By using the source montage in the individual headframe, the mean of the norm of the dipole moment of the hippocampal-associated sources was calculated for the above-mentioned time segment per person and then analyzed statistically.

### Statistics

Statistical analyses were conducted using *R 2.8.1 *[R Development Core [70]] and SPSS (SPSS Statistics 17.0). For assessing age effects on test results (cognitive capacity) and behavioral data (number of training trials needed to reach criterion, number of errors, reaction times), linear regressions were conducted with the constant variable age and dependent variables test results and behavioral data, respectively. For examining effects of age and cognitive competence on hippocampal activity, repeated measurement analyses of covariance (ANCOVA) with the factor hemisphere (left, right) and the covariates age and neuropsychological test results were conducted. In order to capture possible nonlinearities, restricted cubic spline transformations of age were investigated [71], but they did not improve model fit as measured by AIC [72] and were discarded.

## Results

### Neuropsychological test performance

Significant effects of age on cognitive performance were found for several tests. Elderly people performed worse in the digit symbol test, the digit span test, Benton test, Boston naming test, Mini Mental State Examination, word list learning, word list delayed recall, figure recall, trail making test version A and version B. No significant effects of age were found for verbal fluency, figure drawing as well as word list recognition (for details see Table 1).

**Table 1:** Influence of age on neuropsychological test performance

Dependent Variable (neuropsychological tests)	β - Coefficient	*F*	*p*
Digit Symbol	-.67	*F*(1,50) = 41.18	< .0001*
Digit Span	-.28	*F*(1,50) = 4.30	.04*
Benton-corr	-.64	*F*(1,50) = 33.88	< .0001*
Boston Naming Test	-.38	*F*(1,50) = 8.40	.01*
MMSE	-.35	*F*(1,50) = 7.00	.01*
WLL	-.47	*F*(1,50) = 13.78	.001*
WLDR	-.43	*F*(1,50) = 11.33	.001*
WLR	-.23	*F*(1,50) = 2.90	.10
FD	-.22	*F*(1,50) = 2.60	.11
FR	-.49	*F*(1,50) = 16.06	< .0001*
VF	-.12	*F*(1,50) = .74	.39
TMT-A	.46	*F*(1,50) = 13.69	.001*
TMT-B	.51	*F*(1,50) = 17.34	< .0001*

Influence of age on neuropsychological test performance (linear regression with constant variable age and dependent variables test results). Digit Symbol = Digit Symbol subtest of the German version of the Wechsler Adult Intelligence Scale; Digit Span = Digit Span subtest of the German version of the Wechsler Adult Intelligence Scale; Benton-corr = correct answers of the Benton Visual Retention Test (revised form); MMSE = Mini Mental State Examination; WLL = Subtest Word List-Learning of the German version of the CERAD-NP-Plus test battery; WLDR = Subtest Word List-Delayed Recall of the German version of the CERAD-NP-Plus test battery; WLR = Subtest Word List-Recognition of the German version of the CERAD-NP-Plus test battery; FD = Subtest Figure Drawing of the German version of the CERAD-NP-Plus test battery; FR = Subtest Figure Recall of the German version of the CERAD-NP-Plus test battery; VF = Sum score verbal fluency (semantic and phonemic) of the German version of the CERAD-NP-Plus test battery; TMT-A = Trail Making Test - Version A; TMT-B = Trail Making Test - Version B

* p < .05

### Behavioral performance during the Transverse Patterning task

There was no age effect on the number of training trials needed to reach criterion or on the number of incorrect trials during MEG measurement. Only reaction times were significantly longer for elderly people (Table 2).

**Table 2:** Influence of age on behavioral performance in the transverse patterning task

	TP		
	
Dependent Variable (behavioral data)	β	*F*	*p*
Training trials	.24	F(1,29) = 1.72	.20
Incorrect trials	.06	F(1,50) = .17	.69
Reaction times	.44	F(1,50) = 11.69	.001*

Influence of age on behavioral performance (linear regression with constant variable age and dependent variables behavioral data). Training trials = number of training trials needed to reach criterion; Incorrect responses = number of incorrect trials during MEG measurement; Reaction times = Reaction times for the correct trials

*p < .05

### Neuromagnetic data

**Figure 5:**
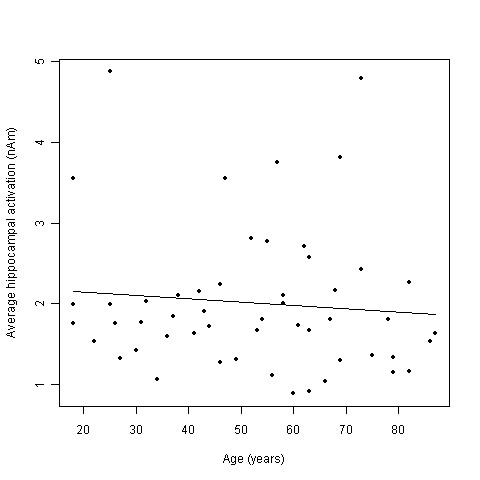
**Correlation of the average hippocampal activation (nAm) and age (years)**.

For the selected time window of 130-220 ms after stimulus presentation, subjects showed a mean source strength of 2.18 nAm (*SD *= 1.19) for the left and 1.97 nAm (*SD *= .99) for the right hemisphere. Statistical analyses did not show significant effects of age (*F*(1,50) = .41; *p *= .53) (see Figure 5), sex (*F*(1,50) = .27; *p *= .61) or hemisphere (*F*(1,51) = 1.94; *p *= .17) on the mean amplitudes of the hippocampal sources.

#### Influence of cognitive competence on brain activity

**Figure 6:**
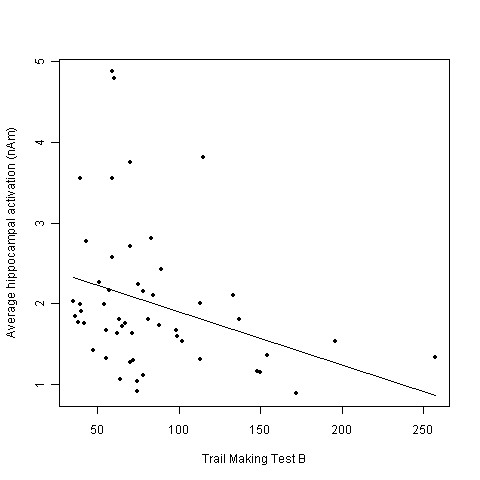
**Correlation of hippocampal activation and performance in Trail Making Test - B**. Correlation of the average hippocampal activation (nAm) and the performance in the Trail Making Test - B (higher number means worse performance).

There were significant relationships between several neuropsychological test results and hippocampal activation (see Table 3). The influence of two verbal memory tests were positive, i.e. higher scores in word list learning (*p *= .03) and word list delayed recall (*p *= .04) were associated with higher hippocampal activity (β_(WLL) _= .08; β_(WLDR) _= .14). Additionally, higher scores in both versions of the trail making test (which means worse performance) were associated with lower hippocampal activity (*p*_(TMT-A) _= .03; β_(TMT-A) _= -.02; *p*_(TMT-B) _= .045; β_(TMT-B)_= -.01) (Figure 6).

**Table 3:** Influence of test performance and age on mean amplitude of the hippocampal sources

Covariate: Testresults				Covariate: Age		
	
	*β*-Coefficient	*F*	*p*	*β*-Coefficient	*F*	*p*
Digit Symbol	.02	*F*(1,49) = 3.29	.08	.006	*F*(1,49) = .43	.52
Digit Span	.01	*F*(1,49) = .14	.72	-. 003	*F*(1,49) = .40	.53
Benton-corr	-.04	*F*(1,49) = 1.18	.28	-.01	*F*(1,49) = .41	.53
VF	.03	*F*(1,49) = 3.12	.08	- .003	*F*(1,49) = .43	.52
WLL	.08	*F*(1,49) = 4.79	.03*	.03	*F*(1,49) = .44	.51
WLDR	.14	*F*(1,49) = 4.68	.04*	.002	*F*(1,49) = .44	.51
WLR	.19	*F*(1,49) = 1.18	.28	- .002	*F*(1,49) = .41	.53
FR	- .006	*F*(1,49) = .01	.91	- .005	*F*(1,49) = .40	.53
TMT-A	-.02	*F*(1,49) = 5.16	.03*	.003	*F*(1,49) = .44	.51
TMT-B	-.007	*F*(1,49) = 4.2	.045*	.003	*F*(1,49) = .44	.51

Influence of test performance and age on mean amplitude of the hippocampal sources (repeated measurement ANCOVA, factor hemisphere). Digit Symbol = Digit Symbol subtest of the German version of the Wechsler Adult Intelligence Scale; Digit Span = Digit Span subtest of the German version of the Wechsler Adult Intelligence Scale; Benton-corr = correct answers of the Benton Visual Retention Test (revised form); VF = Sum score verbal fluency (semantic and phonemic) of the German version of the CERAD-NP-Plus test battery; WLL = Subtest Word List-Learning of the German version of the CERAD-NP-Plus test battery; WLDR = Subtest Word List-Delayed Recall of the German version of the CERAD-NP-Plus test battery; WLR = Subtest Word List-Recognition of the German version of the CERAD-NP-Plus test battery; FR = Subtest Figure Recall of the German version of the CERAD-NP-Plus test battery; TMT-A = Trail Making Test - Version A; TMT-B = Trail Making Test - Version B

* p < .05

Furthermore, there were statistical trends of positive relationships between the digit symbol test and hippocampal activity (*p *= .08; β_(Digit Symbol) _= .02) and verbal fluency and hippocampal activity (*p *= .08; β_(VF) _= .03). The relationship between the covariates digit span test, Benton test, word list recognition as well as figure recall with hippocampal activity did not reach significance.

## Discussion

This study examined whether hippocampal activity during the transverse patterning task varies with age and whether it is also modulated by cognitive competence. Our study yielded two main findings: First, no age effect on hippocampal activity was observed. Second, hippocampal activity was associated with cognitive competence. The strengths and limitations of this study are summarized in table 4.

**Table 4:** Strengths and limitations of the study

Strengths and implications
The study indicates that in non-pathological aging, hippocampal neural activity does not necessarily decrease with age but is rather related to cognitive competence. These results challenge the "myth" that age decreases hippocampal activity.
The study comprised a remarkably big sample (N = 52) for neuroimaging studies and the subject pool spans a large age range (18-89 years) and includes middle-aged participants that many age-related studies lack.
A very comprehensive battery of neuropsychological tests was applied leading to a well-characterized sample.

**Limitations**

As this is the first study, future studies need to replicate this finding.
A co-registration of MR- and MEG-activity would have been desireable, but in this study no MR scans from participants were available. Therefore, the detected activity might not have originated from the hippocampus directly but from adjacent temporal lobe structures. Future studies should combine structural and functional brain imaging techniques.

### Age-related effects on hippocampal activity

Young and elderly people showed a similar pattern of hippocampal source strength. This is in line with a number of studies that also did not find age-related differences in the extent of hippocampal activation during memory tasks [22,23,28,31-33,73]. These studies provide evidence of intact hippocampal activation during healthy aging, which is consistent with our findings. Many other studies assessing neuronal activity during memory tasks, however, found either more [20,29,30] or less [19,21,24-27] activity in the hippocampal region for elderly people. They typically investigated brain activation during memory tasks that activate a wide network of different brain areas and therefore it is not surprising that the results are inconsistent. In this study however, we used a hippocampus specific task and neither did we observe age-related changes in hippocampal current flow nor age-related differences in behavioral performance. At first glance this seems to be contradictory to the age-related deficits in behavioral performance found by Driscoll and colleagues [45]. However, the lack of age-related differences in our study could be explained by differences in experimental setup: Driscoll et al. (2003) used a stepwise approach where subjects had to detect the rules on their own by trial and error which is most likely more difficult than learning the rules by explicit instructions. Given that older adults show longer periods of task acquisition and lower mastery than their younger counterparts with increasing task difficulty [e.g. [74]], it is not surprising that the performance of the elderly subjects was inferior to the performance of the young group in the Driscoll (2003) study. In our study, participants learned the rule by explicit instruction and except for the reaction times, we did not observe age-related deficits in the performance on the transverse patterning task. Since young and elderly performed similarly well, age-related differences in activation of hippocampal-associated components could not necessarily be expected.

Hippocampal activation seems to differ between healthy elderly people and people with mild cognitive impairment (MCI) or Alzheimer's disease (AD). While we did not find age-related differences in hippocampal activation in our healthy sample, it was reported that people with AD show decreased hippocampal activity [31,75], whereas people with MCI paradoxically show more hippocampal activation [32,75]. Less impaired people with MCI show a pattern of hippocampal hyperactivation, whereas more impaired persons demonstrate hippocampal hypoactivation [32]. Miller and colleagues [76] found that greater hippocampal activation during memory tasks in people with MCI serves as a predictor for greater subsequent cognitive decline. Furthermore, they suggested that hippocampal hyperactivation may represent an attempted compensatory response to accumulating neurodegenerative pathology. In addition, Dickerson and colleagues [77] hypothesized that increased hippocampal activation in MCI may serve as a marker for impending clinical decline. In fact, plaques disrupt the synchrony of convergent inputs and thus may lead to an increased number of failures for signal transmission [78]. This in turn may lead to larger rather than smaller PSPs and with it to a smaller intracellular current flow (which produces the MEG signal).

One further explanation for our results could be that we had a cognitively still highly fit sample: there was neither a significant effect of age on the number of training trials needed to reach criterion, nor on the number of errors made during MEG measurement. Furthermore, we merely investigated the correct trials. As could be expected, elderly people responded significantly slower than younger people but besides this, young and elderly people performed similarly well. Therefore, age-related changes in neuronal activation patterns could not necessarily be expected.

### Effects of cognitive competence on hippocampal activation

Our data reveal a significant relation between cognitive competence and hippocampal source strength: better performance in tests for verbal memory, executive functioning, verbal fluency, and cognitive speed was associated with higher hippocampal activity, whereas worse performance in executive functions (higher scores in the Trail Making test) was related to lower hippocampal activity. As mentioned above, several studies exist which examine age-related hippocampal activation during different experimental tasks and related performance in these tasks [e.g. [19-24,27-33,73]]. However, to our knowledge, this study is the first to examine the correlation between hippocampal activity and independently assessed general cognitive capacity. Results indicate that there might be a more general relationship between medial temporal lobe activity and cognitive performance, with higher general cognitive competence being associated with higher hippocampal activity. This is also in line with the findings from the MCI and AD studies mentioned above [31,32,75-77]: the more cognitively impaired the participants were in these studies, the lower was their hippocampal activation. Further studies are needed to clarify and confirm the significance of these findings.

### Hippocampal activation in MEG

Results indicate that MEG is a useful method to investigate functional changes in deeper brain structures such as the hippocampus, in line with several earlier studies using similar methods [e.g. [50,51,58,63]]. One limitation of MEG technology, however, is that the detected activity might not have originated from the hippocampus directly but from adjacent temporal lobe structures. However, the transverse patterning task is a well-known paradigm for assessing hippocampal function, which has been demonstrated in many studies (e.g. Astur 1998; Reed and Squire 1999; Hanlon, Weisend et al. 2003; Astur and Constable 2004; Hanlon, Weisend et al. 2005; Meltzer 2007; Moses 2007).

## Conclusions

It is generally assumed that age-related morphological changes in the hippocampus result in functional deficits that contribute to cognitive decline. In the present study we employed the hippocampus-dependent transverse patterning task to assess whether hippocampal activity varies with increasing age and whether it is also associated with cognitive competence. To our knowledge, this study is the first to examine the correlation between hippocampal activity and general cognitive capacity in healthy aging. While one might assume that hippocampal activity would decrease with increasing age, we found no such effect in healthy aging. In addition, our results suggest that hippocampal neural activity during the transverse patterning task is associated with cognitive competence. In sum, our results support the notion that in non-pathological aging, hippocampal neural activity does not necessarily decrease with age but is rather related to cognitive competence.

## Authors' contributions

VML, ITK and TE designed the experiment. VML collected the data and performed the MEG data analysis with support of CW and IPJ. SK performed the statistical analyses. VML drafted the manuscript and ITK, CW, SK, IPJ as well as TE revised it critically. All authors commented on and approved the final manuscript.

## Supplementary Material

Additional file 1 **Sample description**. The table illustrates each volunteer's age, sex and associated diseases. Click here for file

## References

[B1] Liu RS Lemieux L Bell GS Sisodiya SM Shorvon SD Sander JW Duncan JS A longitudinal study of brain morphometrics using quantitative magnetic resonance imaging and difference image analysis Neuroimage 2003 20 1 22 33 10.1016/S1053-8119(03)00219-2 14527567

[B2] Fotenos AF Snyder AZ Girton LE Morris JC Buckner RL Normative estimates of cross-sectional and longitudinal brain volume decline in aging and AD Neurology 2005 64 6 1032 1039 15781822 10.1212/01.WNL.0000154530.72969.11

[B3] Coffey CE Wilkinson WE Parashos IA Soady SA Sullivan RJ Patterson LJ Figiel GS Webb MC Spritzer CE Djang WT Quantitative cerebral anatomy of the aging human brain: a cross-sectional study using magnetic resonance imaging Neurology 1992 42 3 Pt 1 527 536 1549213 10.1212/wnl.42.3.527

[B4] Jernigan TL Press GA Hesselink JR Methods for measuring brain morphologic features on magnetic resonance images. Validation and normal aging Arch Neurol 1990 47 1 27 32 2294890 10.1001/archneur.1990.00530010035015

[B5] Pfefferbaum A Mathalon DH Sullivan EV Rawles JM Zipursky RB Lim KO A quantitative magnetic resonance imaging study of changes in brain morphology from infancy to late adulthood Arch Neurol 1994 51 9 874 887 8080387 10.1001/archneur.1994.00540210046012

[B6] Skullerud K Variations in the size of the human brain. Influence of age, sex, body length, body mass index, alcoholism, Alzheimer changes, and cerebral atherosclerosis Acta Neurol Scand Suppl 1985 102 1 94 3887832

[B7] Brown J Cooper-Kuhn CM Kempermann G Van Praag H Winkler J Gage FH Kuhn HG Enriched environment and physical activity stimulate hippocampal but not olfactory bulb neurogenesis Eur J Neurosci 2003 17 10 2042 2046 10.1046/j.1460-9568.2003.02647.x 12786970

[B8] Raz N Lindenberger U Rodrigue KM Kennedy KM Head D Williamson A Dahle C Gerstorf D Acker JD Regional brain changes in aging healthy adults: general trends, individual differences and modifiers Cereb Cortex 2005 15 11 1676 1689 10.1093/cercor/bhi044 15703252

[B9] Burke SN Barnes CA Neural plasticity in the ageing brain Nat Rev Neurosci 2006 7 1 30 40 10.1038/nrn1809 16371948

[B10] Resnick SM Pham DL Kraut MA Zonderman AB Davatzikos C Longitudinal magnetic resonance imaging studies of older adults: a shrinking brain J Neurosci 2003 23 8 3295 3301 12716936 10.1523/JNEUROSCI.23-08-03295.2003 PMC6742337

[B11] Salat DH Buckner RL Snyder AZ Greve DN Desikan RS Busa E Morris JC Dale AM Fischl B Thinning of the cerebral cortex in aging Cereb Cortex 2004 14 7 721 730 10.1093/cercor/bhh032 15054051

[B12] Sutherland RJ Rudy JW Configural association theory: The role of the hippocampal formation in learning, memory, and amnesia Psychobiology 1989 17 2 129 144

[B13] Cohen NJ Eichenbaum H Memory, amnesia, and the hippocampal system 1993 Cambridge: MA US: The MIT Press

[B14] Raz N Rodrigue KM Head D Kennedy KM Acker JD Differential aging of the medial temporal lobe: a study of a five-year change Neurology 2004 62 3 433 438 14872026 10.1212/01.wnl.0000106466.09835.46

[B15] Raz N Rodrigue KM Kennedy KM Acker JD Hormone replacement therapy and age-related brain shrinkage: regional effects Neuroreport 2004 15 16 2531 2534 10.1097/00001756-200411150-00020 15538189

[B16] Raz N Gunning-Dixon F Head D Rodrigue KM Williamson A Acker JD Aging, sexual dimorphism, and hemispheric asymmetry of the cerebral cortex: replicability of regional differences in volume Neurobiol Aging 2004 25 3 377 396 10.1016/S0197-4580(03)00118-0 15123343

[B17] Malykhin NV Bouchard TP Camicioli R Coupland NJ Aging hippocampus and amygdala Neuroreport 2008 19 5 543 547 10.1097/WNR.0b013e3282f8b18c 18388735

[B18] Geula C Abnormalities of neural circuitry in Alzheimer's disease: hippocampus and cortical cholinergic innervation Neurology 1998 51 1 Suppl 1 S18 29 discussion S65-17 9674759 10.1212/wnl.51.1_suppl_1.s18

[B19] Murty VP Sambataro F Das S Tan HY Callicott JH Goldberg TE Meyer-Lindenberg A Weinberger DR Mattay VS Age-related Alterations in Simple Declarative Memory and the Effect of Negative Stimulus Valence J Cogn Neurosci 2009 21 10 1920 1933 10.1162/jocn.2009.21130 18823239 PMC2757312

[B20] Rajah MN McIntosh AR Age-related differences in brain activity during verbal recency memory Brain Res 2008 1199 111 125 10.1016/j.brainres.2007.12.051 18282558

[B21] Dennis NA Hayes SM Prince SE Madden DJ Huettel SA Cabeza R Effects of aging on the neural correlates of successful item and source memory encoding J Exp Psychol Learn Mem Cogn 2008 34 4 791 808 10.1037/0278-7393.34.4.791 18605869 PMC2752883

[B22] Morcom AM Good CD Frackowiak RS Rugg MD Age effects on the neural correlates of successful memory encoding Brain 2003 126 Pt 1 213 229 10.1093/brain/awg020 12477708

[B23] Duverne S Motamedinia S Rugg MD The Relationship between Aging, Performance. and the Neural Correlates of Succesful Memory Encoding Cereb Cortex 2009 19 3 733 744 10.1093/cercor/bhn122 18653664 PMC2637304

[B24] Gutchess AH Welsh RC Hedden T Bangert A Minear M Liu LL Park DC Aging and the neural correlates of successful picture encoding: frontal activations compensate for decreased medial-temporal activity J Cogn Neurosci 2005 17 1 84 96 10.1162/0898929052880048 15701241

[B25] Park DC Welsh RC Marshuetz C Gutchess AH Mikels J Polk TA Noll DC Taylor SF Working memory for complex scenes: age differences in frontal and hippocampal activations J Cogn Neurosci 2003 15 8 1122 1134 10.1162/089892903322598094 14709231

[B26] Cabeza R Daselaar SM Dolcos F Prince SE Budde M Nyberg L Task-independent and task-specific age effects on brain activity during working memory, visual attention and episodic retrieval Cereb Cortex 2004 14 4 364 375 10.1093/cercor/bhg133 15028641

[B27] Dennis NA Kim H Cabeza R Age-related differences in brain activity during true and false memory retrieval J Cogn Neurosci 2008 20 8 1390 1402 10.1162/jocn.2008.20096 18303982 PMC3114875

[B28] Miller SL Celone K DePeau K Diamond E Dickerson BC Rentz D Pihlajamaki M Sperling RA Age-related memory impairment associated with loss of parietal deactivation but preserved hippocampal activation Proc Natl Acad Sci USA 2008 105 6 2181 2186 10.1073/pnas.0706818105 18238903 PMC2538895

[B29] Restom K Bangen KJ Bondi MW Perthen JE Liu TT Cerebral blood flow and BOLD responses to a memory encoding task: a comparison between healthy young and elderly adults Neuroimage 2007 37 2 430 439 10.1016/j.neuroimage.2007.05.024 17590353 PMC2214854

[B30] Maguire EA Frith CD Aging affects the engagement of the hippocampus during autobiographical memory retrieval Brain 2003 126 Pt 7 1511 1523 10.1093/brain/awg157 12805116

[B31] Sperling RA Bates JF Chua EF Cocchiarella AJ Rentz DM Rosen BR Schacter DL Albert MS fMRI studies of associative encoding in young and elderly controls and mild Alzheimer's disease J Neurol Neurosurg Psychiatry 2003 74 1 44 50 10.1136/jnnp.74.1.44 12486265 PMC1738201

[B32] Celone KA Calhoun VD Dickerson BC Atri A Chua EF Miller SL DePeau K Rentz DM Selkoe DJ Blacker D Alterations in memory networks in mild cognitive impairment and Alzheimer's disease: an independent component analysis J Neurosci 2006 26 40 10222 10231 10.1523/JNEUROSCI.2250-06.2006 17021177 PMC6674636

[B33] Rand-Giovannetti E Chua EF Driscoll AE Schacter DL Albert MS Sperling RA Hippocampal and neocortical activation during repetitive encoding in older persons Neurobiol Aging 2006 27 1 173 182 10.1016/j.neurobiolaging.2004.12.013 16298252

[B34] Bontempi B Laurent-Demir C Destrade C Jaffard R Time-dependent reorganization of brain circuitry underlying long-term memory storage Nature 1999 400 6745 671 675 10.1038/23270 10458162

[B35] Amidzic O Riehle HJ Fehr T Wienbruch C Elbert T Pattern of focal gamma-bursts in chess players Nature 2001 412 6847 603 10.1038/35088119 11493907

[B36] Spence KW The nature of the response in discrimination learning Psychol Rev 1952 59 1 89 93 10.1037/h0063067 14912194

[B37] Alvarado MC Rudy JW Rats with damage to the hippocampal-formation are impaired on the transverse-patterning problem but not on elemental discriminations Behav Neurosci 1995 109 2 204 211 10.1037/0735-7044.109.2.204 7619311

[B38] Astur RS Configural learning in humans: The transverse patterning problem Psychobiology 1998 26 3 176 182

[B39] Rickard TC Grafman J Losing their configural mind. Amnesic patients fail on transverse patterning J Cogn Neurosci 1998 10 4 509 524 10.1162/089892998562915 9712680

[B40] Reed JM Squire LR Impaired transverse patterning in human amnesia is a special case of impaired memory for two-choice discrimination tasks Behav Neurosci 1999 113 1 3 9 10.1037/0735-7044.113.1.3 10197901

[B41] Rickard TC Verfaellie M Grafman J Transverse patterning and human amnesia J Cogn Neurosci 2006 18 10 1723 1733 10.1162/jocn.2006.18.10.1723 17014376 PMC1698463

[B42] Alvarado MC Selective Neurotoxic Damage to the Hippocampal Formation Impairs Performance of the Transverse Patterning and Location Memory in Rhesus Macaques Hippocampus 2005 15 1 118 131 10.1002/hipo.20037 15390158

[B43] Alvarado MC Bachevalier J Comparison of the effects of damage to the perirhinal and parahippocampal cortex on transverse patterning and location memory in rhesus macaques J Neurosci 2005 25 6 1599 1609 10.1523/JNEUROSCI.4457-04.2005 15703414 PMC6725995

[B44] Alvarado MC Wright AA Bachevalier J Object and spatial relational memory in adult rhesus monkeys is impaired by neonatal lesions of the hippocampal formation but not the amygdaloid complex Hippocampus 2002 12 4 421 433 10.1002/hipo.1115 12201627

[B45] Driscoll I Hamilton DA Petropoulos H Yeo RA Brooks WM Baumgartner RN Sutherland RJ The aging hippocampus: cognitive, biochemical and structural findings Cereb Cortex 2003 13 12 1344 1351 10.1093/cercor/bhg081 14615299

[B46] Breier JI Simos PG Zouridakis G Papanicolaou AC Relative timing of neuronal activity in distinct temporal lobe areas during a recognition memory task for words J Clin Exp Neuropsychol 1998 20 6 782 790 10.1076/jcen.20.6.782.1116 10484690

[B47] Breier JI Simos PG Zouridakis G Papanicolaou AC Lateralization of cerebral activation in auditory verbal and non-verbal memory tasks using magnetoencephalography Brain Topogr 1999 12 2 89 97 10.1023/A:1023458110869 10642008

[B48] Gonsalves BD Kahn I Curran T Norman KA Wagner AD Memory strength and repetition suppression: multimodal imaging of medial temporal cortical contributions to recognition Neuron 2005 47 5 751 761 10.1016/j.neuron.2005.07.013 16129403

[B49] Hamada Y Sugino K Kado H Suzuki R Magnetic fields in the human hippocampal area evoked by a somatosensory oddball task Hippocampus 2004 14 4 426 433 10.1002/hipo.10196 15224980

[B50] Hanlon FM Weisend MP Huang M Lee RR Moses SN Paulson KM Thoma RJ Miller GA Canive JM A non-invasive method for observing hippocampal function Neuroreport 2003 14 15 1957 1960 10.1097/00001756-200310270-00015 14561928

[B51] Hanlon FM Weisend MP Yeo RA Huang M Lee RR Thoma RJ Moses SN Paulson KM Miller GA Canive JM A specific test of hippocampal deficit in schizophrenia Behav Neurosci 2005 119 4 863 875 10.1037/0735-7044.119.4.863 16187815

[B52] Ioannides AA Liu MJ Liu LC Bamidis PD Hellstrand E Stephan KM Magnetic field tomography of cortical and deep processes: examples of "real-time mapping" of averaged and single trial MEG signals Int J Psychophysiol 1995 20 3 161 175 10.1016/0167-8760(95)00031-3 8788219

[B53] Martin T McDaniel MA Guynn MJ Houck JM Woodruff CC Bish JP Moses SN Kicic D Tesche CD Brain regions and their dynamics in prospective memory retrieval: a MEG study Int J Psychophysiol 2007 64 3 247 258 10.1016/j.ijpsycho.2006.09.010 17126436

[B54] Mikuni N Nagamine T Ikeda A Terada K Taki W Kimura J Kikuchi H Shibasaki H Simultaneous recording of epileptiform discharges by MEG and subdural electrodes in temporal lobe epilepsy Neuroimage 1997 5 4 Pt 1 298 306 10.1006/nimg.1997.0272 9345559

[B55] Moses SN Ryan JD Bardouille T Kovacevic N Hanlon FM McIntosh AR Semantic information alters neural activation during transverse patterning performance Neuroimage 2009 46 3 863 873 10.1016/j.neuroimage.2009.02.042 19281852 PMC2789295

[B56] Nishitani N Nagamine T Fujiwara N Yazawa S Shibasaki H Cortical-hippocampal auditory processing identified by magnetoencephalography J Cogn Neurosci 1998 10 2 231 247 10.1162/089892998562672 9555109

[B57] Papanicolaou AC Simos PG Castillo EM Breier JI Katz JS Wright AA The hippocampus and memory of verbal and pictorial material Learn Mem 2002 9 3 99 104 10.1101/lm.44302 12074997 PMC182590

[B58] Riggs L Moses SN Bardouille T Herdman AT Ross B Ryan JD A complementary analytic approach to examining medial temporal lobe sources using magnetoencephalography Neuroimage 2009 45 2 627 642 10.1016/j.neuroimage.2008.11.018 19100846

[B59] Stephen JM Ranken DM Aine CJ Weisend MP Shih JJ Differentiability of simulated MEG hippocampal, medial temporal and neocortical temporal epileptic spike activity J Clin Neurophysiol 2005 22 6 388 401 16462195 10.1097/01.WNP.0000172141.26081.78

[B60] Tendolkar I Rugg M Fell J Vogt H Scholz M Hinrichs H Heinze HJ A magnetoencephalographic study of brain activity related to recognition memory in healthy young human subjects Neurosci Lett 2000 280 1 69 72 10.1016/S0304-3940(99)01001-0 10696814

[B61] Tesche CD Non-invasive detection of ongoing neuronal population activity in normal human hippocampus Brain Res 1997 749 1 53 60 10.1016/S0006-8993(96)01286-3 9070627

[B62] Tesche CD Karhu J Theta oscillations index human hippocampal activation during a working memory task Proc Natl Acad Sci USA 2000 97 2 919 924 10.1073/pnas.97.2.919 10639180 PMC15431

[B63] Moses SN Ryan JD Bardouille T Kovacevic N Hanlon FM McIntosh AR Semantic information alters neural activation during transverse patterning performance Neuroimage 2009 46 3 863 873 10.1016/j.neuroimage.2009.02.042 19281852 PMC2789295

[B64] Ackenheil M Stotz G Dietz-Bauer R Vossen AI M.I.N.I. 5.0.0. German version/DSM-IV 1999 Munich: Psychiatrische Universitätsklinik

[B65] Oldfield R The assessment and analysis of handedness: the Edinburgh inventory Neuropsychologia 1971 9 1 97 113 10.1016/0028-3932(71)90067-4 5146491

[B66] Morris JC Mohs RC Rogers H Fillenbaum G Heyman A Consortium to establish a registry for Alzheimer's disease (CERAD) clinical and neuropsychological assessment of Alzheimer's disease Psychopharmacol Bull 1988 24 4 641 652 3249766

[B67] Tewes U Hamburg-Wechsler-Intelligenztest für Erwachsene (HAWIE-R) 1991 Bern: Verlag Hans Huber

[B68] Steck PH A revision of A. L. Benton's Visual Retention Test (BVRT) in two parallel forms Archives of Clinical Neuropsychology 2005 20 409 416 10.1016/j.acn.2004.09.009 15797176

[B69] Scherg M Bast T Hoechstetter K Ille N Weckesser D Bornfleth H Berg P Brain source montages improve the non-invasive diagnosis in epilepsy International Congress Series 2004 1270 15 19 10.1016/j.ics.2004.04.035

[B70] Team RDC R: A language and environment for statistical computing (version 2.8.1) 2008 Vienna, Austria: R Foundation for Statisitcal Computing

[B71] Harrell FE Regression Modelling Strategies 2001 New York: Springer

[B72] Akaike H Petrov BN, Caski S Information theory and an extension of the maximum likelihood principle2nd International Symposium on Information Theory: 1973; Akademiai Kiado 1973 267 281

[B73] Abram K Teplin L Charles D Longworth S McClelland G Dulcan M Posttraumatic stress disorder and trauma in youth in juvenile detention Archives of General Psychiatry 2004 61 4 403 410 10.1001/archpsyc.61.4.403 15066899 PMC2861915

[B74] Fisk AD Rogers WA Cooper BP Gilbert DK Automatic category search and its transfer: aging, type of search, and level of learning J Gerontol B Psychol Sci Soc Sci 1997 52 2 P91 102 9060984 10.1093/geronb/52b.2.p91

[B75] Sperling R Functional MRI studies of associative encoding in normal aging, mild cognitive impairment, and Alzheimer's disease Ann N Y Acad Sci 2007 1097 146 155 10.1196/annals.1379.009 17413017

[B76] Miller SL Fenstermacher E Bates J Blacker D Sperling RA Dickerson BC Hippocampal activation in adults with mild cognitive impairment predicts subsequent cognitive decline J Neurol Neurosurg Psychiatry 2008 79 6 630 635 10.1136/jnnp.2007.124149 17846109 PMC2683145

[B77] Dickerson BC Salat DH Bates JF Atiya M Killiany RJ Greve DN Dale AM Stern CE Blacker D Albert MS Medial temporal lobe function and structure in mild cognitive impairment Ann Neurol 2004 56 1 27 35 10.1002/ana.20163 15236399 PMC4335689

[B78] Stern EA Bacskai BJ Hickey GA Attenello FJ Lombardo JA Hyman BT Cortical synaptic integration in vivo is disrupted by amyloid-beta plaques J Neurosci 2004 24 19 4535 4540 10.1523/JNEUROSCI.0462-04.2004 15140924 PMC6729398

