# Supplementary material for: Hippocampal activity during the transverse patterning task declines with cognitive competence but not with age
Source: BMC Neurosci. 2010 Sep 8;11:113. doi: 10.1186/1471-2202-11-113 (PMC2944355; doi:10.1186/1471-2202-11-113)
Supplement: Additional file 1 — Sample description. The table illustrates each volunteer's age, sex and associated diseases. [file 1471-2202-11-113-S1.DOC]

Supplements

| **Subject Number** | **Age (years)** | **Sex (m/f)** | **Diseases** |
| --- | --- | --- | --- |
| 1 | 57 | m | - |
| 2 | 63 | m | - |
| 3 | 25 | m | - |
| 4 | 87 | f | Rheumatism, hypertension |
| 5 | 44 | f | - |
| 6 | 41 | f | - |
| 7 | 79 | f | Hypertension |
| 8 | 42 | f | - |
| 9 | 58 | f | - |
| 10 | 55 | f | Hypothyroidism |
| 11 | 78 | f | - |
| 12 | 49 | f | - |
| 13 | 56 | m | - |
| 14 | 58 | f | Hyperthyroidism |
| 15 | 36 | m | - |
| 16 | 30 | m | - |
| 17 | 63 | m | - |
| 18 | 46 | f | - |
| 19 | 27 | f | - |
| 20 | 61 | f | - |
| 21 | 53 | f | - |
| 22 | 62 | f | - |
| 23 | 67 | m | - |
| 24 | 25 | f | - |
| 25 | 69 | m | Hypothyroidism |
| 26 | 79 | m | Hypertension |
| 27 | 60 | m | - |
| 28 | 73 | m | - |
| 29 | 63 | f | - |
| 30 | 66 | f | - |
| 31 | 31 | m | - |
| 32 | 46 | m | Hypothyroidism |
| 33 | 37 | m | - |
| 34 | 32 | m | - |
| 35 | 47 | f | - |
| 36 | 54 | f | - |
| 37 | 86 | f | - |
| 38 | 26 | m | - |
| 39 | 68 | f | High cholesterol level |
| 40 | 73 | f | Hypertension, High cholesterol level |
| 41 | 18 | m | - |
| 42 | 18 | m | - |
| 43 | 43 | m | - |
| 44 | 52 | m | - |
| 45 | 34 | f | - |
| 46 | 38 | f | - |
| 47 | 69 | f | - |
| 48 | 82 | f | Hypothyroidism |
| 49 | 82 | m | Hypertension |
| 50 | 75 | m | Hypertension |
| 51 | 22 | f | - |
| 52 | 18 | f | - |
